# Genetic determinants of risk in autoimmune pulmonary alveolar proteinosis

**DOI:** 10.1038/s41467-021-21011-y

**Published:** 2021-02-15

**Authors:** Saori Sakaue, Etsuro Yamaguchi, Yoshikazu Inoue, Meiko Takahashi, Jun Hirata, Ken Suzuki, Satoru Ito, Toru Arai, Masaki Hirose, Yoshinori Tanino, Takefumi Nikaido, Toshio Ichiwata, Shinya Ohkouchi, Taizou Hirano, Toshinori Takada, Satoru Miyawaki, Shogo Dofuku, Yuichi Maeda, Takuro Nii, Toshihiro Kishikawa, Kotaro Ogawa, Tatsuo Masuda, Kenichi Yamamoto, Kyuto Sonehara, Ryushi Tazawa, Konosuke Morimoto, Masahiro Takaki, Satoshi Konno, Masaru Suzuki, Keisuke Tomii, Atsushi Nakagawa, Tomohiro Handa, Kiminobu Tanizawa, Haruyuki Ishii, Manabu Ishida, Toshiyuki Kato, Naoya Takeda, Koshi Yokomura, Takashi Matsui, Masaki Watanabe, Hiromasa Inoue, Kazuyoshi Imaizumi, Yasuhiro Goto, Hiroshi Kida, Tomoyuki Fujisawa, Takafumi Suda, Takashi Yamada, Yasuomi Satake, Hidenori Ibata, Nobuyuki Hizawa, Hideki Mochizuki, Atsushi Kumanogoh, Fumihiko Matsuda, Koh Nakata, Tomomitsu Hirota, Mayumi Tamari, Yukinori Okada

**Affiliations:** 1 Department of Statistical Genetics, Osaka University Graduate School of Medicine, Suita, Japan; 2 Department of Allergy and Rheumatology, Graduate School of Medicine, the University of Tokyo, Tokyo, Japan; 3 Center for Data Sciences, Harvard Medical School, Boston, USA; 4 Divisions of Genetics and Rheumatology, Department of Medicine, Brigham and Women’s Hospital, Harvard Medical School, Boston, USA; 5 Program in Medical and Population Genetics, Broad Institute of MIT and Harvard, Cambridge, USA; 6 Division of Respiratory Medicine and Allergology, Department of Internal Medicine, School of Medicine, Aichi Medical University, Aichi, Japan; 7 Clinical Research Center, National Hospital Organization Kinki-Chuo Chest Medical Center, Sakai, Osaka, Japan; 8 Center for Genomic Medicine, Kyoto University Graduate School of Medicine, Kyoto, Japan; 9 Pharmaceutical Discovery Research Laboratories, TEIJIN PHARMA LIMITED, Hino, Japan; 10 Department of Pulmonary Medicine, Fukushima Medical University, Fukushima, Japan; 11 Department Respiratory Medicine, Tokyo Medical University, Tokyo, Japan; 12 Occupational Health, Graduate School of Medicine, Tohoku University, Miyagi, Japan; 13 Respiratory Medicine, School of Medicine, Tohoku University, Miyagi, Japan; 14 Uonuma Institute of Community Medicine, Niigata University Medical and Dental Hospital, Niigata, Japan; 15 Department of Neurosurgery, Faculty of Medicine, the University of Tokyo, Tokyo, Japan; 16 Department of Respiratory Medicine and Clinical Immunology, Osaka University Graduate School of Medicine, Suita, Japan; 17 Laboratory of Immune Regulation, Department of Microbiology and Immunology, Osaka University Graduate School of Medicine, Suita, Japan; 18 Department of Otorhinolaryngology - Head and Neck Surgery, Osaka University Graduate School of Medicine, Suita, Japan; 19 Department of Neurology, Osaka University Graduate School of Medicine, Suita, Japan; 20 Department of Obstetrics and Gynecology, Osaka University Graduate School of Medicine, Suita, Japan; 21 Department of Pediatrics, Osaka University Graduate School of Medicine, Suita, Japan; 22 Student Support and Health Administration Organization, Tokyo Medical and Dental University, Tokyo, Japan; 23 Department of Clinical Medicine, Institute of Tropical Medicine, Nagasaki University, Nagasaki, Japan; 24 Department of Infectious Diseases, Nagasaki University Hospital, Nagasaki University, Nagasaki, Japan; 25 Department of Respiratory Medicine, Faculty of Medicine and Graduate School of Medicine, Hokkaido University, Sapporo, Japan; 26 Department of Respiratory Medicine, Kobe City Medical Center General Hospital, Kobe, Japan; 27 Department of Advanced Medicine for Respiratory Failure, Graduate School of Medicine, Kyoto University, Kyoto, Japan; 28 Department of Respiratory Medicine, Graduate School of Medicine, Kyoto University, Kyoto, Japan; 29 Department of Respiratory Medicine, Kyorin University, Mitaka, Japan; 30 Department of Respiratory Medicine and Allergology, Kariya Toyota General Hospital, Kariya, Japan; 31 Department of Respiratory Medicine, Respiratory Disease Center, Seirei Mikatahara General Hospital, Hamamatsu, Japan; 32 Department of Pulmonary Medicine, Graduate School of Medical & Dental Sciences, Kagoshima University, Kagoshima, Japan; 33 Department of Respiratory Medicine, Fujita Health University School of Medicine, Aichi, Japan; 34 Department of Respiratory Medicine, National Hospital Organization Osaka Toneyama Medical Center, Toyonaka, Japan; 35 Second Division, Department of Internal Medicine, Hamamatsu University School of Medicine, Hamamatsu, Japan; 36 Department of Respiratory Medicine, Shizuoka City Shizuoka Hospital, Shizuoka, Japan; 37 Department of Respiratory Medicine, National Hospital Organization Mie Chuo Medical Center, Tsu, Japan; 38 Department of Pulmonary Medicine, Faculty of Medicine, University of Tsukuba, Tsukuba, Japan; 39 Laboratory of Immunopathology, World Premier International Immunology Frontier Research Center (WPI-IFReC), Osaka University, Suita, Japan; 40 Integrated Frontier Research for Medical Science Division, Institute for Open and Transdisciplinary Research Initiatives, Osaka University, Suita, Japan; 41 Division of Advanced Medical Development, Niigata University Medical and Dental Hospital, Niigata, Japan; 42 Division of Molecular Genetics, the Jikei University School of Medicine, Research Center for Medical Science, Tokyo, Japan; 43 Laboratory of Statistical Immunology, World Premier International Immunology Frontier Research Center (WPI-IFReC), Osaka University, Suita, Japan

**Keywords:** Genome-wide association studies, Respiratory tract diseases

## Abstract

Pulmonary alveolar proteinosis (PAP) is a devastating lung disease caused by abnormal surfactant homeostasis, with a prevalence of 6–7 cases per million population worldwide. While mutations causing hereditary PAP have been reported, the genetic basis contributing to autoimmune PAP (aPAP) has not been thoroughly investigated. Here, we conducted a genome-wide association study of aPAP in 198 patients and 395 control participants of Japanese ancestry. The common genetic variant, rs138024423 at 6p21, in the major-histocompatibility-complex (MHC) region was significantly associated with disease risk (Odds ratio [OR] = 5.2; *P* = 2.4 × 10^−12^). HLA fine-mapping revealed that the common HLA class II allele, HLA-DRB1*08:03, strongly drove this signal (OR = 4.8; *P* = 4.8 × 10^−12^), followed by an additional independent risk allele at HLA-DPβ1 amino acid position 8 (OR = 0.28; *P* = 3.4 × 10^−7^). HLA-DRB1*08:03 was also associated with an increased level of anti-GM-CSF antibody, a key driver of the disease (β = 0.32; *P* = 0.035). Our study demonstrated a heritable component of aPAP, suggesting an underlying genetic predisposition toward an abnormal antibody production.

## Abstract (web summary)

Autoimmune pulmonary alveolar proteinosis (aPAP) is a complex lung disease caused by abnormal surfactant homeostasis. Here, the authors carry out a genome-wide association study of aPAP in a Japanese cohort, finding variants in the MHC and suggesting predisposition to abnormal antibody production.

## Introduction

Pulmonary alveolar proteinosis (PAP) is a rare diffuse lung disease with a prevalence of 6–7 cases per million population worldwide^1,2^. The disease is characterized by the abnormal accumulation of pulmonary surfactant within pulmonary alveoli, resulting in progressive respiratory failure and increased infection risk^2–5^. PAP has three distinct etiologies: hereditary, autoimmune, and secondary^6^. Approximately 90–95% of cases of PAP are of autoimmune etiology, in which a high level of autoantibodies against granulocyte–macrophage colony-stimulating factor (GM-CSF) neutralize the biologic activity of GM-CSF^7^, thereby causing poor surfactant clearance. While rare mutations in the GM-CSF receptor α or β chains (*CSF2RA*, *CSF2RB*)^8^, the surfactant proteins B or C^9^, ATP-binding cassette subfamily A member 3 (*ABCA3*)^10^, and thyroid transcription factor-1^11^ were identified as causing hereditary PAP, the genetic basis underlying autoimmune PAP (aPAP) has never been thoroughly investigated due to its low prevalence. In particular, given a growing evidence that common genetic variants in major-histocompatibility-complex (MHC) region are associated with rare autoimmune diseases^12–14^, an investigation of human leukocyte antigen (HLA) alleles associated with the risk of aPAP and the production of autoantibodies against GM-CSF is warranted.

Here, we perform a genome-wide association study (GWAS) of aPAP in the Japanese population to investigate the genetic basis in the risk of aPAP. We further perform HLA imputation to fine map the association signal within the MHC locus. We show a significant association of an HLA allele with the risk of aPAP, as well as increased levels of anti-GM-CSF antibody.

## Results

### Study populations

We studied a total of 198 patients with aPAP of Japanese ancestry and 395 control participants of Japanese ancestry. The patients were enrolled through a nation-wide collaborative recruitment strategy^2,15^. The patient characteristics are summarized in Table 1. All patients were confirmed to have a positive serum anti-GM-CSF antibody level.

**Table 1:** Clinical characteristics of the patients and the controls at baseline.

Variable	PAP patients (*N* = 198)	Control participants (*N* = 395)
Age—yr	57.4 ± 12.9	48.7 ± 21.3
Female sex—no. (%)	79 (40)	185 (47)
Autoantibodies against GM-CSF—μg/ml	69.3 ± 80.2	N/A

Plus–minus values are means ± standard deviation.

### Single-marker association test

**Fig. 1:**
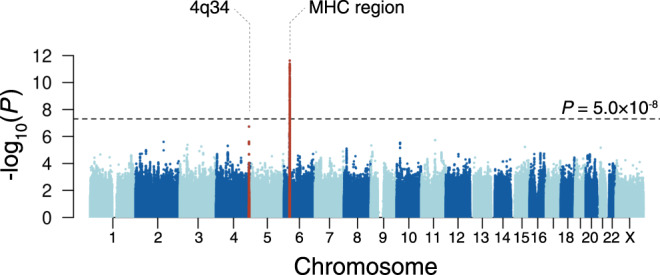
Manhattan plot for the genome-wide association study of autoimmune pulmonary alveolar proteinosis. A Manhattan plot showing −log_10_(*P*) of the genome-wide single-marker association test of aPAP in the Japanese population. The dotted horizontal line represents a genome-wide significance threshold of *P* = 5.0 × 10^–8^. The loci that satisfied the genome-wide significance threshold are colored in red. MHC, major histocompatibility complex.

We performed a one-stage GWAS with all the case samples and controls together to maximize the statistical power, given the low prevalence of the disease. We analyzed 12,153,232 genetic markers that passed the stringent post-imputation quality control threshold (Rsq > 0.7). The MHC locus was identified as associated with the aPAP risk with a genome-wide significance (*P* < 5.0 × 10^−8^; Fig. 1; Supplementary Table 1). The lead variant was rs138024423, where the G allele (deletion) was strongly associated with the increased disease risk (Odds ratio [OR], 5.2; 95% confidence interval [CI], 3.3 to 8.2; *P* = 2.4 × 10^−12^). The frequency of G allele was higher in cases than in controls (the allele frequency = 0.21 and 0.072, respectively). We additionally identified the risk variant with suggestive significance at 4q34 (rs56125424; OR, 3.0; 95% CI, 2.0–4.6; *P* = 1.9 × 10^−7^; Supplementary Fig. 1 for a regional plot). This region is relatively devoid of known genes, and the suggestive variant rs56125424 is located in the intergenic region between the LINC01098 and LINC00290 (1.8 Mb and 1.3 Mb away, respectively). The previously reported genetic associations of this region (i.e., the variants located within 1 Mb from rs56125424) with human complex traits included body mass index^16–18^, waist-to-hip ratio^16^, hand-grip strength^19^, smoking initiation^20^, general risk tolerance^21^, and motion sickness^22^. No autoimmune diseases have been reported to date according to the GWAS catalog^23^. There was no interaction effect between rs138024423 at MHC locus and rs56125424 at 4q34 on the risk of aPAP (*P* = 0.70).

### MHC risk fine-mapping by HLA imputation

**Fig. 2:**
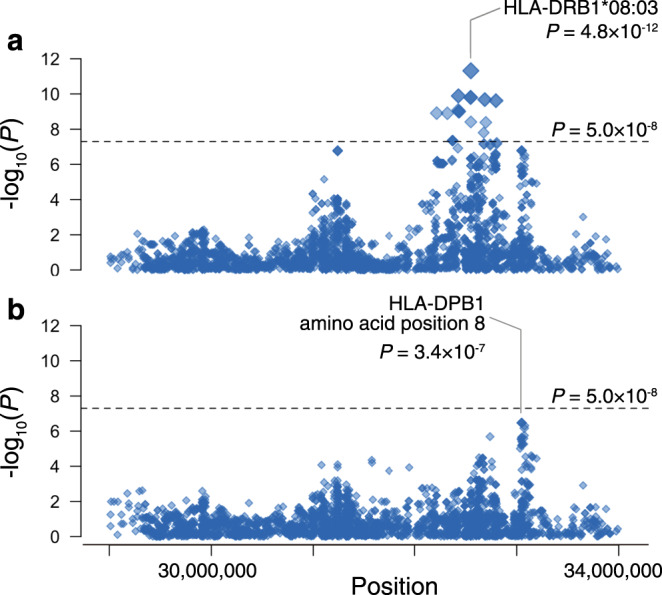
Regional associations of the variants in the major histocompatibility complex (MHC) region with autoimmune pulmonary alveolar proteinosis risk. Regional associations of the variants in the MHC region with aPAP risk based on the HLA imputation analysis. **a** Each diamond represents the −log_10_(*P*) of association of the variants, including the single nucleotide polymorphism, two-digit and four-digit HLA alleles, and amino acid polymorphisms of HLA genes. The dotted horizontal line represents the genome-wide significance threshold of *P* = 5.0 × 10^–8^. The lead HLA allele is labeled. **b** Each diamond represents the –log_10_(*P*) of the secondary associations after conditioning on the dosage of the lead variant HLA-DRB1*08:03. The dotted horizontal line represents the genome-wide significance threshold of *P* = 5.0 × 10^–8^.

To fine map the disease risk within the complex MHC region^24,25^, we applied HLA imputation method to the genotype data using a high-resolution reference panel of 1120 individuals of Japanese ancestry^25^. After the stringent post-imputation quality control, we obtained genotype dosages of 101 two-digit, 164 four-digit, and 180 six-digit HLA alleles and 1701 amino acid polymorphisms of classical and nonclassical HLA genes in the entire MHC region. We assessed the association of the imputed HLA alleles and amino acid polymorphisms with the risk of aPAP. We found that the common HLA class II allele, HLA-DRB1*08:03, strongly drove the signal in the MHC region (Fig. 2a; OR, 4.8; 95% CI, 3.1–7.5; *P* = 4.8 × 10^−12^). The frequency of this allele was 0.22 in cases, whereas 0.074 in controls. To rule out the potential uncontrolled bias caused by the heterogeneous distribution of the HLA alleles according to the geographical location, we conducted a sensitivity analysis by stratifying the whole cohort into two datasets based on the location of the recruitment centers, Set 1 and Set 2 (see “Methods”). We observed that the effect sizes of both datasets were consistent with the primary association (OR, 5.6 and 4.7, respectively; Supplementary Fig. 2), and thus concluded that uncontrolled biases should be minimal. We also confirmed that a suggestive signal at 4q34 had similar effect sizes in stratified analyses as well (OR, 3.6 and 3.9, respectively; Supplementary Fig. 3).

To identify additional MHC associations independent of *HLA-DRB1*, we conditioned on HLA-DRB1*08:03 and tested again the HLA alleles and amino acid polymorphisms. We observed an independent association signal at position 8 of the amino acid polymorphisms in *HLA-DPB1* (Fig. 2b; *P* = 3.4 × 10^−7^). At this position, the amino acid residue Val8 was protective against aPAP when compared with the amino acid residue Leu8 (OR, 0.28; 95% CI, 0.17–0.46). After conditioned on HLA-DRB1*08:03 and HLA-DPβ1 amino acid position 8, no additional MHC associations were observed (*P* > 2.8 × 10^−5^).

Given the driver role of the neutralizing anti-GM-CSF antibody in the pathogenesis of aPAP, we finally sought to investigate the association of the risk HLA alleles with serum anti-GM-CSF antibody levels. We observed that the lead risk HLA allele, HLA-DRB1*08:03, was positively associated with the increased level of anti-GM-CSF antibody (effect size = 0.32, *P* = 0.035) within the cases of aPAP. Although the statistical power in the within-case analysis is limited if we consider the multiple testing, this might suggest that HLA-DRB1*08:03 is genetically associated with the increased production of anti-GM-CSF antibody, thereby predisposing individuals harboring this allele to the increased risk of developing aPAP.

The allele frequency of HLA-DRB1*08:03 is common in Asian population (e.g., 8.3% in Japanese, 5.8% in Chinese, and 5.4% in Vietnamese), but very rare in other populations including Europeans (e.g., 0% in British, 0.29% in German, 0% in Italian, and 0.24% in European Caucasian in USA) according to the Allele Frequency Net Database^26^. The frequency heterogeneity was replicated in the UK Biobank resource of global populations^27^. Previous reports regarding the genetic association of HLA-DRB1*08:03 with human diseases and traits are thus limited to the Asian populations. Chen et al.^28^ reported that HLA-DRB1*08:03 was associated with increased risk of drug-induced agranulocytosis in patients with Graves’ disease (OR, 4.36, *P* = 1.8 × 10^−9^) in Taiwanese. While not reaching the genome-wide significance threshold, there had been a suggestive association of HLA-DRB1*08:03 with primary biliary cholangitis in the Japanese population (OR, 1.75, *P* = 1.0 × 10^−7^)^29^, and one with systemic lupus erythematosus (SLE) in the Korean population (OR, 1.59, *P* = 7.4 × 10^−8^)^30^. Intriguingly, although there exists no systematic epidemiological observation of the comorbidity of aPAP and these autoimmune diseases, there have been two case reports of comorbidity of SLE and aPAP^31,32^. We speculated that the Asian specific allele of HLA-DRB1*08:03 might have a shared etiological basis for abnormal self- or drug-derived antigen presentation and production of autoantibodies.

## Discussion

In this study, we provided evidence of significant genetic contribution to the pathogenesis of aPAP. The strong genetic risk was identified within the MHC region. We further fine mapped the association signals, and revealed that HLA-DRB1*08:03 significantly drove the genetic risk, followed by an additional risk at HLA-DPβ1 amino acid position 8. We finally showed that this allele was also associated with the increased production of anti-GM-CSF antibody, a key driver of the disease. The effect size of the identified genetic risk was much larger than those of any of the previously suggested epidemiological risk factors for aPAP, such as cigarette smoking.

Given its autoimmune etiology, the association of variants in the MHC region with the risk of aPAP has long been expected, while no significant association had been reported to date^33^. Our study provided the first evidence of HLA association in the susceptibility to aPAP with genome-wide significance, which was enabled by a nation-wide collaborative patient recruitment strategy and a clear diagnostic criterion for enrollment^2,5^. This effort led to the recruitment and genotyping of around one fourth of the estimated patients with PAP in Japan. We performed the one-stage GWAS in this study because of the extremely low prevalence of the disease, which hampered the recruitment of completely independent patients as a replication dataset. In addition, the lead allele, HLA-DRB1*08:03, is Asian-specific, which makes a replication in other populations difficult. Although we mitigated a risk of spurious associations caused by population stratification by conducting stratified analyses based on the geographical location of recruitment centers, a future GWAS in other Asian cohorts is warranted to confirm the replicability of the association. Furthermore, GWAS in other populations would elucidate the comprehensive genetic architecture of the disease across ancestries.

Intriguingly, the lead HLA allele that strongly increased the risk of the disease was common (MAF > 5%) in the Japanese population, despite the extremely low prevalence of the disease. We speculated that the risk variants within HLA class II alleles led to the presentation of self-peptide of GM-CSF to immunocompetent cells. Previously, Uchida et al.^34^ reported that anti-GM-CSF antibodies were detected in healthy individuals but in far lower level than in aPAP, and that anti-GM-CSF autoantibodies were associated with impaired GM-CSF-dependent myeloid functions at levels above a critical threshold. Our results showing that the disease risk allele of HLA-DRB1*08:03 was also associated with an increased level of anti-GM-CSF antibodies that might support the hypothetical pathogenesis of aPAP. Further analyses incorporating rare variants from whole-genome sequencing (WGS) or environmental interactions might decipher the pathogenetic architecture of this disease.

The aPAP is characterized by large variations in disease severity and clinical prognosis. For patients with severe symptoms, approved treatments are limited to whole lung lavage. The clinical outcome of patients with comorbidities is unsatisfactory^35^. Of note, a randomized control trial recently showed a significant effect on the laboratory outcome of inhaled rhGM-CSF (sargramostim) in patients with aPAP but no clinically important changes in outcomes in a limited sample size^15^. The current guidelines for diagnosis and treatment of PAP would have a room for improvement, by optimization for individual clinical outcome and accounting for novel treatment options^5^. Our study might suggest a potential clinical utility of genetic risk in explaining the differences in the severity or treatment response and in constructing efficient treatment strategies.

## Methods

### Study populations

Patients with PAP were recruited from major hospitals throughout Japan as a nationwide collaborative project. We enrolled patients with a diagnosis of aPAP based on findings on high-resolution computed tomography (CT) and biopsy, cytologic findings on bronchoalveolar lavage, or both, with a positive serum anti-GM-CSF antibody level (>1.0 μg per milliliter) (see Supplementary Methods). Serum anti-GM-CSF antibody levels were measured by ELISA as described elsewhere^36^. Control participants were recruited at Osaka University or related institutions (see Supplementary Methods). All participants provided written informed consent approved by the institutional review board of each participating hospital or institution. This study was approved by the ethical committee of Aichi Medical University, the Jikei University School of Medicine, and Osaka University.

### Genotyping and quality control

Hundred and ninety-eight patients with aPAP and 395 control participants were genotyped using Infinium Asian Screening Array. This genotyping array was built using an East Asian reference panel including WGS, which enabled efficient genotyping in East Asian populations.

For sample quality control (QC), we excluded samples with low genotyping call rates (call rate <98%) and in close genetic relation (PI_HAT calculated by PLINK^37^ > 0.175). We included samples of the estimated East Asian ancestry, based on the principal component analysis with the samples of HapMap project^38^ (Supplementary Fig. 4). We confirmed that no sample had the heterozygosity rate greater than the mean + 3 SD of all the individuals. For variant QC, we excluded variants meeting any of the following criteria: (1) genotyping call rate < 98%, (2) *P* value for Hardy–Weinberg equilibrium < 1.0 × 10^−6^, and (3) minor allele count < 5. In addition we excluded variants with >10% frequency difference with the imputation reference panel. This QC process yielded 498,097 autosomal and 16,207 X chromosomal scaffold variants.

### Whole-genome imputation

We used shapeit2 software^39^ for haplotype phasing with the use of haplotype reference. After the phasing, we used Minimac3 software^40^ for genotype imputation. As an imputation reference, we used the reference haplotypes of 1000 Genomes Project Phase 3 version 5 genotype (*n* = 2,504) and Japanese WGS data (*n* = 1037)^41^, which were recently constructed and validated for imputation accuracy^42^. We used imputed variants with Rsq > 0.7 in the association analysis.

### Genome-wide association study

We performed GWAS by using logistic regression to test single-marker genetic associations with the risk of aPAP on the basis of imputed allelic dosage. We included age, sex, and top 20 principal components as covariates to account for potential population structure. We used genetically determined sex as a covariate. We considered a genome-wide significance threshold in the association as *P* < 5.0 × 10^−8^. We set a suggestive significance threshold and a significance threshold in a conditional analysis as *P* < 5.0 × 10^−7^. We assessed the genotyping accuracy of the lead SNP and the suggestive SNP (i.e., rs138024423 at MHC locus and rs56125424 at 4q34) by comparing the SNP-array based genotype with WGS data for part of the control individuals (*n* = 181). We confirmed that the concordance between the SNP-array based genotype and the WGS based genotype was high (99.5% and 100%, respectively).

### HLA imputation and association test

We imputed classical and non-classical HLA alleles (2-, 4-, and 6-digits) and corresponding amino acid sequences using the reference panel recently constructed from 1120 individuals of Japanese ancestry^25^. Briefly, we encoded all variants in the reference panel as biallelic markers. We extracted the genotyped variants in the entire MHC region (24–36 Mb on chromosome 6, NCBI Build 37). Using these variants as a scaffold, we imputed the classical and non-classical HLA alleles (2-, 4-, and 6-digits) and corresponding amino acid sequences using SNP2HLA software^43^. We applied post-imputation quality control to keep the imputed variants with minor allele frequency (MAF) ≥ 0.5% and Rsq > 0.7. For these binary makers that indicated the presence or absence of an investigated HLA allele or an amino acid sequence, we tested an association with the risk of aPAP, using an additive logistic regression model. We included the same covariates as in the primary GWAS. For each marker, we used probabilistic genotypes that took any uncertainty in imputation into account.

### Sensitivity analysis by stratifying the cohort based on the geographical location of recruitment centers

We split the study cohort into two datasets, based on the geographical location of recruitment centers as a sensitivity analysis. The Set 1 included 84 cases from Aichi Medical University and National Hospital Organization Kinki-Chuo Chest Medical Center (located in the west part of Japan) and 252 controls from the west part of Japan. The Set 2 included the remaining 114 cases and 143 controls from the east part of Japan. We then tested the association of HLA-DRB1*08:03 and rs56125424 with the risk of aPAP within each of the two datasets, using the logistic regression model with the same covariates used for the primary analysis.

### Conditional analysis in the MHC locus

To investigate secondary association signals, we used the same additive logistic regression model, additionally including an allelic dosage of the primary lead signal as a covariate.

### Quantitative association test for autoantibody levels

To assess the contribution of each HLA marker to the quantity of serum anti-GM-CSF antibody within the 198 cases, we tested the association of probabilistic genotypes of HLA alleles or amino acid sequences with the normalized value of serum anti-GM-CSF antibody levels, using an additive linear regression model. For the normalization of serum anti-GM-CSF antibody levels, we performed rank-based inverse normal transformation of the residuals, which we obtained from a linear regression model of log-transformed serum anti-GM-CSF antibody levels adjusted for age, sex, and top 20 principal components. The distribution of anti-GM-CSF antibody levels across patients is shown in Supplementary Fig. 5.

### Reporting summary

Further information on research design is available in the Nature Research Reporting Summary linked to this article.

## Supplementary information

Supplementary Information

Reporting Summary

## Data Availability

We provide an interactive visualization of a Manhattan plot with downloadable GWAS summary statistics at our pheweb.jp website [https://pheweb.jp/pheno/PAP]. The summary statistics are also deposited at the National Bioscience Database Center (NBDC) Human Database with the accession code hum0197.v2.
