# Supplementary material for: Genetic determinants of risk in autoimmune pulmonary alveolar proteinosis
Source: Nat Commun. 2021 Feb 15;12:1032. doi: 10.1038/s41467-021-21011-y (PMC7884840; doi:10.1038/s41467-021-21011-y)
Supplement: Supplementary file 1 — Supplementary Information [file 41467_2021_21011_MOESM1_ESM.pdf]

Supplementary Materials for

Genetic determinants of risk in autoimmune pulmonary alveolar proteinosis.

Sakaue S. et al.

Table of Contents

Supplementary Methods **3**

Supplementary Figures **4**

Supplementary Figure 1. 4

Supplementary Figure 2. 5

Supplementary Figure 3. 6

Supplementary Figure 4. 7

Supplementary Figure 5. 8

Supplementary Tables **9**

Supplementary Table 1. 9

Supplementary References **10**

Supplementary Methods

Recruitment of autoimmune pulmonary alveolar proteinosis patients

In this study, we recruited patients with autoimmune pulmonary alveolar proteinosis (aPAP) in collaboration with a nation-wide network of respiratory physicians[1]. The database of aPAP patients was established through this network of pulmonologists treating PAP patients. We diagnosed and recruited patients with aPAP based on the following criteria[1,2].

1. A shadow consistent with PAP in bilateral lungs on high-resolution CT scan is observed.
2. The following conditions (a) or (b) is satisfied.
   a) The appearance of white turbidity is shown in bronchoalveolar lavage (BAL), and precipitation is formed when left alone. Deposition of a granular amorphous substance stained light green with Papanicolaou staining and foamy macrophage are observed on light microscopy. *
   b) Findings supporting pulmonary alveolar proteinosis are observed histopathologically (transbronchial lung biopsy or surgical lung biopsy).
3. A positive result for serum anti-GM-CSF autoantibody titer is confirmed.

All the three criteria must be fulfilled for participants to be enrolled in this study.

* Regarding the staining method for BAL fluid of 2-(a), there may be cases in which staining was conducted by other methods including Giemsa staining, PAS staining, and Diff-Quick staining, instead of Papanicolaou staining. Even in such cases, the criterion 2-(a) was considered as being satisfied when the findings supporting PAP, such as deposition of a granular amorphous substance and foamy macrophage, were observed on light microscopy.

We finally enrolled the diagnosed aPAP patients who provided us with the written informed consent in each of the medical institutions.

Control participants

All control participants were recruited at Osaka University or related institutions, and provided the informed consent with documents approved by the ethical committees. We confirmed that all the control participants did not have nor have a past medical history of immune-related diseases.

Supplementary Figure 1

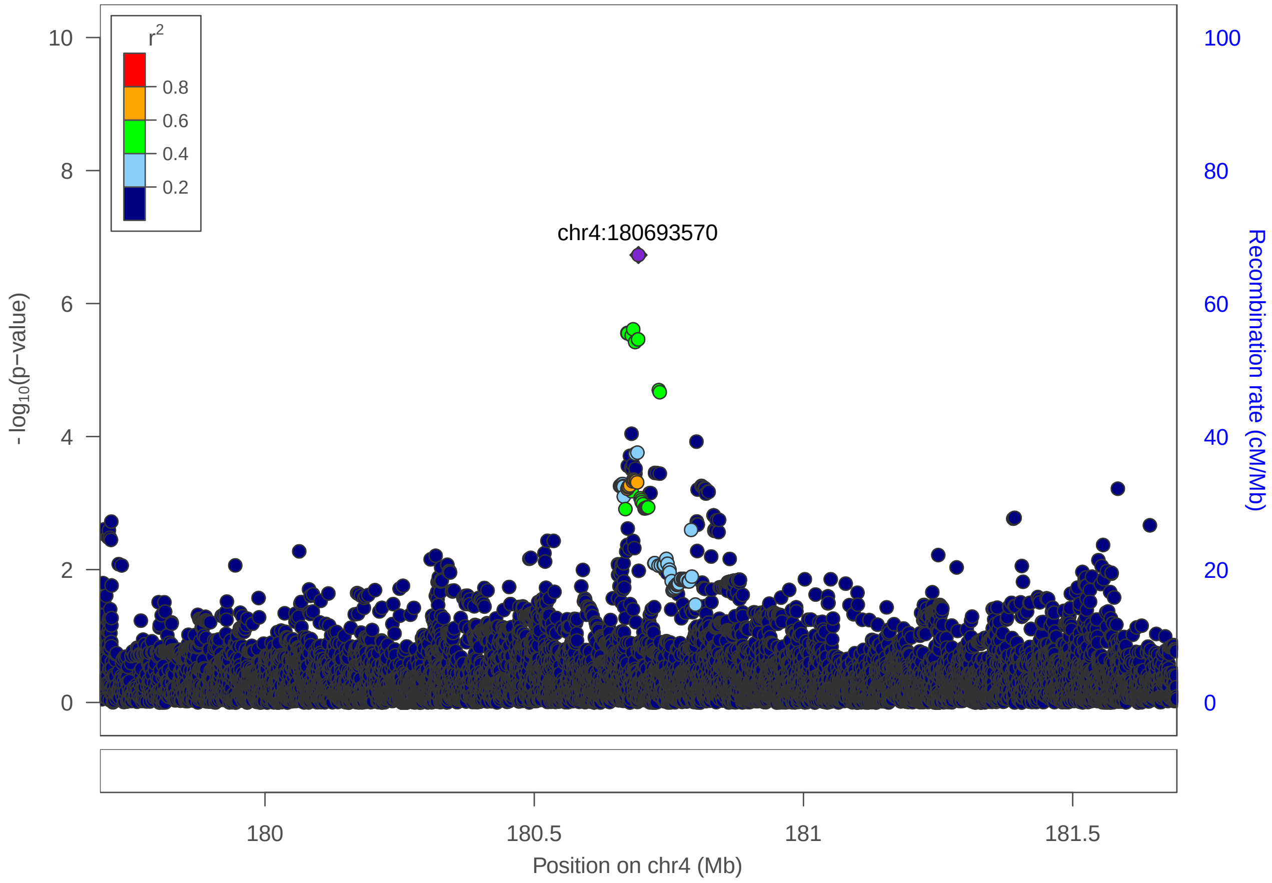

Supplementary Figure 1 | Regional association plot of the suggestive significant loci at 4q34.

A regional association plot of the suggestive significant loci at 4q34 is shown. The *x* axis represents the chromosomal position, and the *y* axis is the –log10 of the *P* value of the single-variant association test. A purple diamond indicates the lead variant, chr4:180693570 (rs56125424). Other circles are colored according to the LD r^2 with this lead variant.

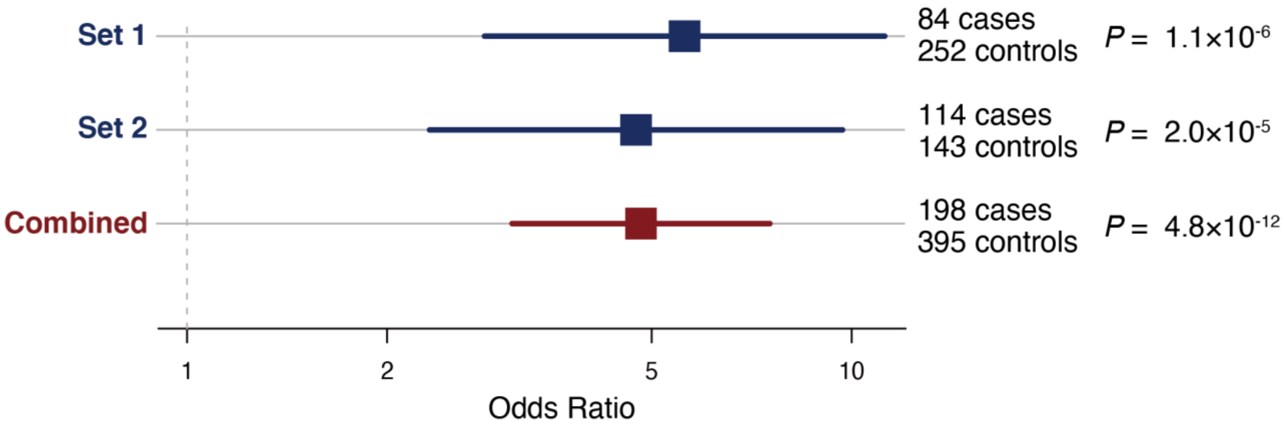

Supplementary Figure 2 | Stratified association study of HLA-DRB1*08:03 based on the recruitment centers.

The association results of the lead HLA allele, HLA-DRB1*08:03, with the risk of pulmonary alveolar proteinosis within Set 1 (mainly from west part of Japan) and Set 2 (mainly from east part of Japan) are shown in blue. The detailed explanation of splitting the cohort is described in **Methods**. In the third row, the association results of HLA-DRB1*08:03 in the whole cohort is shown as a Combined set in red. The boxes indicate the point estimates of the Odds Ratio, and the horizontal bars indicate the 95% confidence interval.

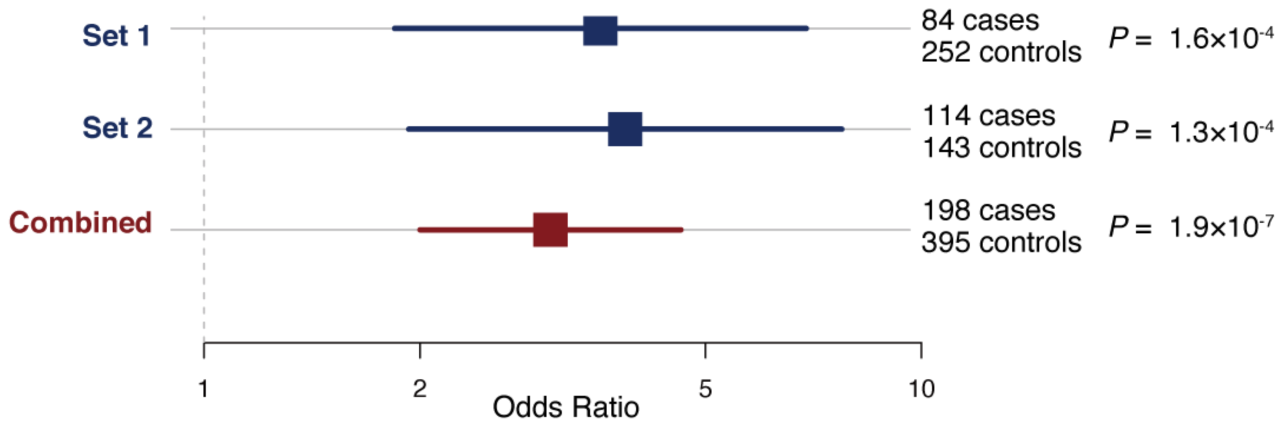

Supplementary Figure 3 | Stratified association study of rs56125424 at 4q34 based on the recruitment centers.

The association results of the variant with a suggestively significance, rs56125424, with the risk of pulmonary alveolar proteinosis within Set 1 (mainly from west part of Japan) and Set 2 (mainly from east part of Japan) are shown in blue. The detailed explanation of splitting the cohort is described in **Methods**. In the third row, the association result of rs56125424 in the whole cohort is shown as a Combined set in red. The boxes indicate the point estimates of the Odds Ratio, and the horizontal bars indicate the 95% confidence interval.

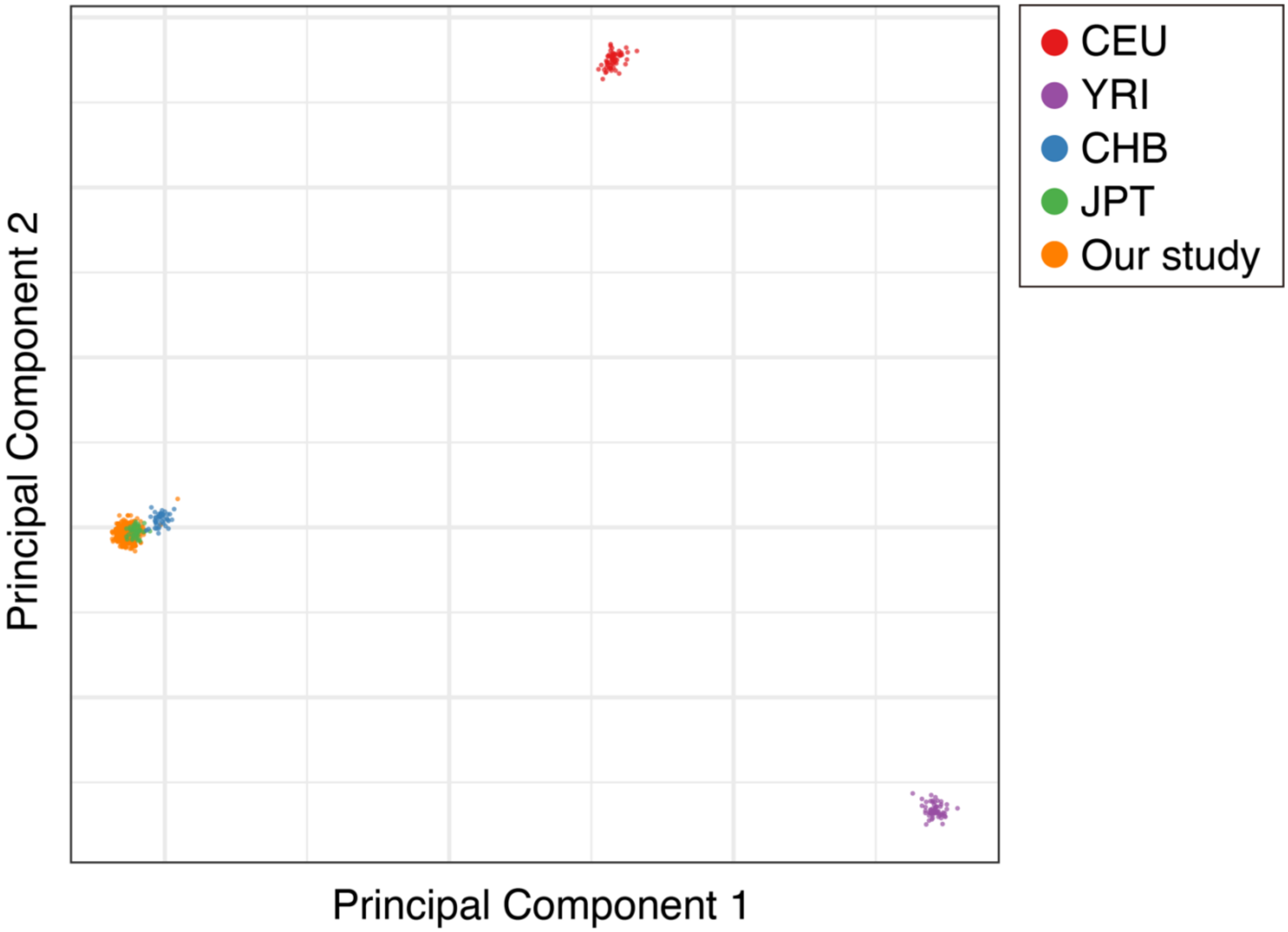

Supplementary Figure 4 | Principal component analysis of the study individuals with the HapMap project individuals.

The first two principal components of the study individuals (in orange) are plotted anchored to the HapMap project individuals of four ancestry. Individuals are colored based on the legend.

CEU, Utah residents with Northern and Western European ancestry; YRI, Yoruba in Ibadan, Nigeria; CHB, Han Chinese in Beijing, China; JPT, Japanese in Tokyo, Japan.

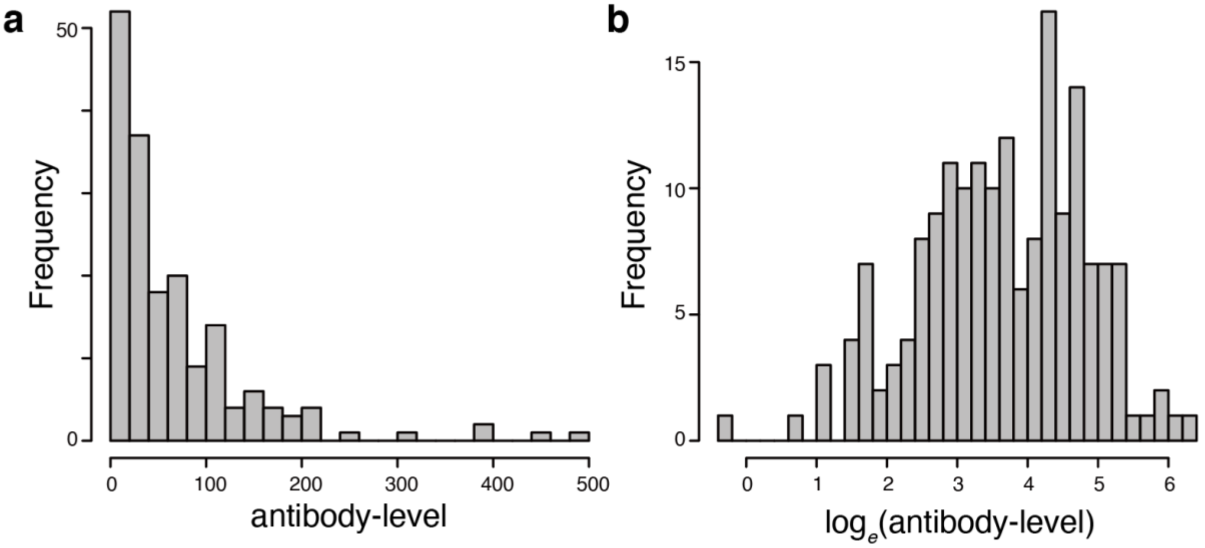

Supplementary Figure 5 | The histogram of anti-GM-CSF antibody levels across patients. The left panel (**a**) shows the distribution of the raw anti-GM-CSF antibody levels, and the right panel (**b**) shows the distribution of the log-transformed anti-GM-CSF antibody levels. The anti-GM-CSF antibody levels are measured in 169 aPAP patients.

Supplementary Table 1

SNP	Chr	Position	Alleles	Coded Allele	Freq_Case	Freq_Control	Odds Ratio	95% CI	*P* Value
rs138024423	6	32453270	GT/G	G	0.21	0.072	5.2	3.3-8.2	2.4×10^{-12}
rs56125424	4	180693570	A/G	G	0.22	0.11	3.0	2.0-4.6	1.9×10^{-7}

Supplementary Table 1 | Summary statistics of the single marker association test.

The statistics of the single-variant association test are shown. Chr; Chromosome. Freq_Case; Frequency in cases. Freq_Control; Frequency in controls. CI; confidence interval.

Supplementary References

1 Inoue Y, Trapnell BC, Tazawa R, *et al.* Characteristics of a large cohort of patients with autoimmune pulmonary alveolar proteinosis in Japan. *Am J Respir Crit Care Med* 2008; **177**: 752–62.

2 Kumar A, Abdelmalak B, Inoue Y, Culver DA. Pulmonary alveolar proteinosis in adults: pathophysiology and clinical approach. *Lancet Respir. Med.* 2018; **6**: 554–65.
