# Supplementary material for: Genetic determinants of risk in autoimmune pulmonary alveolar proteinosis
Source: Nat Commun. 2021 Feb 15;12:1032. doi: 10.1038/s41467-021-21011-y (PMC7884840; doi:10.1038/s41467-021-21011-y)
Supplement: Supplementary file 2 — Reporting Summary [file 41467_2021_21011_MOESM2_ESM.pdf]

Corresponding author(s): Yukinori Okada

Last updated by author(s): Nov 19, 2020

Reporting Summary

Nature Research wishes to improve the reproducibility of the work that we publish. This form provides structure for consistency and transparency in reporting. For further information on Nature Research policies, see our Editorial Policies and the Editorial Policy Checklist.

Statistics

For all statistical analyses, confirm that the following items are present in the figure legend, table legend, main text, or Methods section.

n/a	Confirmed	
☐	☒	The exact sample size (*n*) for each experimental group/condition, given as a discrete number and unit of measurement
☐	☒	A statement on whether measurements were taken from distinct samples or whether the same sample was measured repeatedly
☐	☒	The statistical test(s) used AND whether they are one- or two-sided<br>*Only common tests should be described solely by name; describe more complex techniques in the Methods section.*
☐	☒	A description of all covariates tested
☐	☒	A description of any assumptions or corrections, such as tests of normality and adjustment for multiple comparisons
☐	☒	A full description of the statistical parameters including central tendency (e.g. means) or other basic estimates (e.g. regression coefficient) AND variation (e.g. standard deviation) or associated estimates of uncertainty (e.g. confidence intervals)
☐	☒	For null hypothesis testing, the test statistic (e.g. *F*, *t*, *r*) with confidence intervals, effect sizes, degrees of freedom and *P* value noted<br>*Give P values as exact values whenever suitable.*
☒	☐	For Bayesian analysis, information on the choice of priors and Markov chain Monte Carlo settings
☒	☐	For hierarchical and complex designs, identification of the appropriate level for tests and full reporting of outcomes
☐	☒	Estimates of effect sizes (e.g. Cohen's *d*, Pearson's *r*), indicating how they were calculated

Our web collection on statistics for biologists contains articles on many of the points above.

Software and code

Policy information about availability of computer code

Data collection	No software was used.
Data analysis	We used publicly available software for the data analysis (plink 1.9 and 2.0, shapeit2, Minimac3, and SNP2HLA).

For manuscripts utilizing custom algorithms or software that are central to the research but not yet described in published literature, software must be made available to editors and reviewers. We strongly encourage code deposition in a community repository (e.g. GitHub). See the Nature Research guidelines for submitting code & software for further information.

Data

Policy information about availability of data

All manuscripts must include a data availability statement. This statement should provide the following information, where applicable:
- Accession codes, unique identifiers, or web links for publicly available datasets
- A list of figures that have associated raw data
- A description of any restrictions on data availability

We provide an interactive visualization of a Manhattan plot with downloadable GWAS summary statistics at our pheweb.jp website [https://pheweb.jp/pheno/PAP]. The summary statistics are also deposited at the National Bioscience Database Center (NBDC) Human Database with the accession code hum0197.

Field-specific reporting

Life sciences study design

All studies must disclose on these points even when the disclosure is negative.

Sample size	We recruited 198 patients with pulmonary alveolar proteinosis from major hospitals throughout Japan as a nationwide collaborative project. We enrolled patients with a diagnosis of autoimmune pulmonary alveolar proteinosis based on findings on high-resolution computed tomography (CT) and biopsy, cytologic findings on bronchoalveolar lavage, or both, with a positive serum anti-GM-CSF antibody level (> 1.0 µg per milliliter). This case sample size, which accounted for as many as 25% of the estimated number of patients in Japan and was the largest among genetic studies ever conducted in pulmonary alveolar proteinosis, was successfully powered to genome-wide significant locus.
Data exclusions	We excluded samples with low genotyping call rate, samples in close genetic relation, and ancestry outliers of East Asian population.
Replication	We performed one-stage GWAS given the rarity of the disease but also complementarily performed a sensitivity analysis by stratifying the cohort into two datasets, which showed the consistent result. This sensitivity analyses replicated the genome-wide association at MHC as well as the suggestive association at 4p34.
Randomization	We did not need to use randomization in this study because this is a genotype-phenotype association study. All the samples with available accessibility to genotype and phenotype data were included in the analysis.
Blinding	We did not apply blinding of the samples because this is a genotype-phenotype association study and no intervention was conducted in our study.

Reporting for specific materials, systems and methods

We require information from authors about some types of materials, experimental systems and methods used in many studies. Here, indicate whether each material, system or method listed is relevant to your study. If you are not sure if a list item applies to your research, read the appropriate section before selecting a response.

Materials & experimental systems

n/a	Involved in the study	
☒	☐	Antibodies
☒	☐	Eukaryotic cell lines
☒	☐	Palaeontology and archaeology
☒	☐	Animals and other organisms
☐	☒	Human research participants
☒	☐	Clinical data
☒	☐	Dual use research of concern

Methods

n/a	Involved in the study	
☒	☐	ChIP-seq
☒	☐	Flow cytometry
☒	☐	MRI-based neuroimaging

Human research participants

Policy information about studies involving human research participants

Population characteristics	Case participants are of Japanese ancestry, the mean age was 57, 40% were female, and all of them were tested positive for serum anti-GM-CSF antibody.
Recruitment	We enrolled patients with a diagnosis of autoimmune pulmonary alveolar proteinosis based on findings on high-resolution computed tomography (CT) and biopsy, cytologic findings on bronchoalveolar lavage, or both, with a positive serum anti-GM-CSF antibody level (> 1.0 µg per milliliter) (see the Methods section in the Supplementary Material). All control participants were recruited at Osaka University or related institutions, and provided the informed consent with documents approved by the ethical committees. We confirmed that all the control participants did not have nor have a past medical history of immune-related diseases. We consider a potential bias caused by population stratification, and minimized this bias by incorporating principal components into the regression model. We also confirmed that biases caused by genetic differences due to geographic location of recruitment centers were negligible by conducting stratified analyses.
Ethics oversight	This study was approved by the ethical committee of Aichi Medical University, the Jikei University School of Medicine, and Osaka University.

Note that full information on the approval of the study protocol must also be provided in the manuscript.
